# Failure of daily tenofovir to prevent HIV transmission or the establishment of a significant viral reservoir despite continued antiretroviral therapy

**DOI:** 10.7448/IAS.17.4.19731

**Published:** 2014-11-02

**Authors:** Olubanke Davies, Hannah Alexander, Nicola Robinson, Matthew Pace, Michael Brady, John Frater, Julie Fox

**Affiliations:** 1 HIV, Guys and St Thomas' NHS Trust, London, UK; 2 Sexual Health and HIV, Kings College Hospital, London, UK; 3 Nuffield Department of Medicine, University of Oxford, London, UK; 4 Nuffield Department of Medicine, University of Oxford, Oxford, UK

## Abstract

**Introduction:**

Truvada is licenced for HIV-1 prevention in the USA and is available in the private sector. Tenofovir performed as well as Truvada in the PARTNERS PrEP study and is used as HIV pre-exposure prophylaxis (PreP) in some settings. The clinical efficacy of Tenofovir for PrEP outside a clinical trial is unknown. Antiretroviral therapy (ART) at acute HIV-1 infection (AHI) limits the size of the reservoir, optimizing the chance of maintaining viral control off therapy. As such ART at acute HIV infection is proposed to offer a functional cure in a minority of subjects. We present two cases where Tenofovir PrEP failed to prevent HIV acquisition and failed to limit viral reservoir.

**Materials and Methods:**

Two individuals receiving tenofovir monotherapy for Hepatitis B monoinfection were diagnosed with AHI as defined by a negative HIV antibody test within three months of a positive HIV test following unsafe sex with casual male partners. In-depth histories were taken. Viral genotypes and Tenofovir drug levels were measured from samples taken as close to HIV seroconversion as possible and subsequent samples were analyzed for proviral Total HIV-1 DNA by qPCR.

**Results:**

Patient A had received tenofovir for the preceding six years and always maintained an undetectable Hepatitis B viral load with no concerns about adherence. Two weeks preceding the positive HIV antibody test, he experienced mild symptoms (fever, pharyngitis) of HIV seroconversion. HIV status was confirmed by a repeat fourth generation HIV antibody test and by Western Blot and an HIV viral load was undetectable. Tenofovir trough level at HIV diagnosis was within normal limits. The regimen was intensified to Eviplera and a total HIV-1 DNA was 1381 copies/million CD4 T cells. Patient B received four regimens for hepatitis B treatment before starting tenofovir monotherapy in 2011 and subsequently maintained an undetectable hepatitis B viral load. After three years of tenofovir monotherapy he developed a severe symptomatic seroconversion illness and tested HIV antibody positive. The baseline HIV viral load was 103,306 copies/mL. The regimen was intensified and total HIV-1 DNA was 2746 copies/million CD4 T cells.

**Conclusions:**

Further investigation into the efficacy of tenofovir for PrEP outside a clinical trial is required. ART at AHI does not always lead to a low viral reservoir. To explore the possibility of replication incompetent virus, viral outgrowth assays are underway.

**Table 1:** Summary of the two cases of HIV acquisition whilst receiving tenofovir monotherapy for hepatitis B infection

	Patient A	Patient B
HIV Positive (HIV negative)	14/02/2014 (negative 02/12/2013)	02/06/2014 P24 Ag positive Antibody negative
hepatitis B positive	2008	1997
Hepatitis B treatments	2008 interferon intolerance: 2008 -now Tenofovir	1997 interferon: 2000 3TC; 2002 Famciclovir; 2002–2002 Adefovir monotherapy; 2011 - now Tenofovir
HIV seroconversion symptoms	mild fever 10/12/2013	severe pharyngitis, fever, rash
Baseline HIV viral load copies/ml	<50	158 899
Hepatitis B viral load at HIV diagnosis	undetectable	undetectable
HIV genotype	not possible	wild type
Tenofovir trough level Ng/ml at HIV diagnosis	48 (50th centile is 41)	awaited
Started ART	18/02/2014	06/06/2014
ART regimen	Eviplera	Truvada, Raltegravir, Darunavir, Ritonavir
HIV total DNA copies/million CD4 cells	1381	2746

